# The Implementation Climate for Integrating Buprenorphine Prescribing into Rural Primary Care

**DOI:** 10.1007/s11606-024-09260-1

**Published:** 2024-12-12

**Authors:** Cheyenne Fenstemaker, Elizabeth A. Abrams, Katherine King, Benjamin Obringer, Daniel L. Brook, Vivian Go, William C. Miller, Lindsay Y. Dhanani, Berkeley Franz

**Affiliations:** 1 Ohio University Heritage College of Osteopathic Medicine, Institute to Advance Health Equity, Athens, OH, USA; 2 The Ohio State University College of Medicine, Columbus, OH, USA; 3 Present Address: Department of Psychiatry, University of Colorado Anschutz Medical Campus, Aurora, CO, USA; 4 Gillings School of Public Health, University of North Carolina-Chapel Hill, Chapel Hill, NC, USA; 5 School of Management and Labor Relations, Rutgers University, Piscataway, NJ, USA; 6 Department of Sociology, University of Southern, California Los Angeles, CA, USA

**Keywords:** opioid-related disorders, primary care, implementation, buprenorphine, addiction medicine, rural, rural health

## Abstract

**BACKGROUND:**

Rural communities have been significantly affected by opioid use disorder (OUD) and related harms but have less access to evidence-based medications for opioid use disorder (MOUD), such as buprenorphine. Given the shortage of specialists in these areas, rural primary care is an important setting to expand buprenorphine access, but implementation is limited.

**OBJECTIVE:**

To explore implementation climate factors that support or hinder buprenorphine implementation in rural primary care.

**DESIGN:**

A qualitative study design using in-depth interviews.

**PARTICIPANTS:**

Primary care physicians, nurse practitioners (NPs), and physician associates (PAs) practicing in rural Ohio counties.

**APPROACH:**

Between December 2022 and March 2023, we interviewed participants about their perspectives on buprenorphine prescribing, including using rural primary care as an implementation setting for buprenorphine. Using a deductive, framework-based approach, codes were grouped based on the Consolidated Framework for Implementation Research (CFIR) inner setting factors that contribute to a positive implementation climate for an intervention.

**KEY RESULTS:**

Three implementation climate constructs emerged as decision points for whether to implement buprenorphine in rural primary care: (1) relative priority: the extent to which OUD treatment should be prioritized over other chronic diseases; (2) compatibility: whether buprenorphine prescribing protocols are compatible with the rural primary care setting; (3) tension for change: the extent to which current buprenorphine access shortages in rural communities can be tolerated. Participants expressed mixed perspectives on whether the implementation climate in rural primary care currently supports buprenorphine prescribing.

**CONCLUSION:**

Implementation strategies targeted toward the implementation climate are critical to support buprenorphine prescribing in rural primary care.

## BACKGROUND

Over the past two decades, unintentional overdose rates have increased faster in rural compared to urban areas.^1^ Geographic barriers, social factors, and economic conditions have contributed to increased rates of opioid use disorder (OUD)^2^ and associated infectious disease in rural communities.^3–5^ Treatment for OUD is also more difficult to access in rural regions of the USA, where hospitals have closed^6^ and specialty medical providers are geographically dispersed.^7^ Buprenorphine, one of the three approved medications for OUD (MOUD), reduces mortality and infectious disease morbidity but is greatly underutilized in rural communities with fewer health care professionals willing to prescribe it.^7–9^ Methadone is also more difficult to access as the majority of opioid treatment programs are in urban areas, requiring long commutes for rural residents.^10–13^ Although the X-waiver was removed in late 2022^14^ in an effort to remove barriers to prescribing buprenorphine, adoption has not meaningfully increased.^15^ Thus, without tailored implementation strategies addressing prescribing barriers, rural buprenorphine access disparities will persist.^16,17^

Primary care is a critical site for substance use care in rural communities because specialists are extremely limited.^18^ Primary care is also less stigmatizing than specialty or hospital settings—buprenorphine can be integrated with care for other conditions. This approach normalizes OUD as a chronic disease.^19,20^ Buprenorphine can now be prescribed by physicians, nurse practitioners (NPs), and physician associates (PAs) with decreased federal regulations,^21^ but PCPs remain hesitant to prescribe the medication, and fewer than 8% of family physicians have ever prescribed it.^22^ Providing PCPs with appropriate training and a supportive prescribing environment is essential to expanding buprenorphine access in rural communities.^23^

Many barriers to buprenorphine prescribing^24–31^ have been described in the literature, but strategies to overcome these barriers and increase buprenorphine implementation in primary care are urgently needed. The Consolidated Framework for Implementation Research (CFIR)^32^ is a framework often used to assess barriers and facilitators to the implementation of an evidence-based intervention. Understanding barriers and facilitators specific to different settings is crucial for designing tailored implementation strategies. The CFIR organizes determinants across five domains: (1) outer setting; (2) inner setting; (3) characteristics of individuals; (4) intervention characteristics; and (5) process.

Many implementation strategies to increase buprenorphine adoption have focused on the individual level through PCP education and training or have addressed restrictive prescribing policies in the outer setting. Yet PCPs also report significant inner setting barriers such as limited organizational support for treating addiction in primary care, inadequate time for prescribing buprenorphine, and clinic-level policies that make this work challenging.^33,34^ These barriers may be further exacerbated in rural areas where primary care often fills access gaps for complex disease management, including treating mental health conditions, diabetes, and infectious disease.^35^

One key construct within the inner setting domain is the implementation climate, defined as “the absorptive capacity for change, shared receptivity of involved individuals to an intervention,^36^ and the extent to which use of that intervention will be rewarded, supported, and expected within their organization.”^36^ In other words, the implementation climate refers to whether a certain implementation setting, in this case rural primary care, supports the adoption of an evidence-based intervention such as buprenorphine. The implementation climate construct is made up of six subconstructs that contribute to a positive implementation climate: (1) tension for change; (2) compatibility; (3) relative priority; (4); organizational incentives and rewards; (5) goals and feedback; and (6) learning climate.

The implementation climate is often measured quantitatively using the implementation climate scale, a 38-item measure of organizational-level support for an evidence-based intervention.^37^ Few qualitative studies, however, have explored the different subconstructs that matter for buprenorphine implementation. Among the few studies that do,^38^ rural-specific implementation climate factors have not been examined. A small number of studies have tested models to build practice-level capacity and support for providing buprenorphine in rural primary care.^39–42^ For example, the IT MATTTRs program in rural Colorado used multicomponent implementation strategies at the practice level to support buprenorphine prescribing.^40^ However, existing studies have not explored the role of the implementation climate for buprenorphine prescribing in rural primary care or identified which implementation climate factors are most critical to address.

To better understand the implementation climate^43^ for prescribing buprenorphine within rural primary care, we interviewed PCPs in rural Ohio. Interviews were a part of a clinical trial planning study to develop a buprenorphine prescribing support program for rural primary care practice. Our study contributes to the literature by exploring which implementation climate factors within the rural primary care setting are most likely to shape buprenorphine adoption. As such, these findings will inform implementation strategies to increase buprenorphine access in rural communities by directing health care organizations to the areas most in need of intervention to improve buprenorphine access.

## METHODS

### Study Population and Procedures

Two interviewers (BF and CF), one with doctoral training and one with masters-level training in qualitative methods, conducted semi-structured interviews with PCPs in Ohio between December 2022 and March 2023. We developed the interview guide as part of a broader study which had three phases: (1) exploring barriers and facilitators to prescribing buprenorphine to guide the development of a prescribing support program; (2) iteratively refining the prescribing support program through pilot testing; and (3) a clinical trial measuring implementation of the prescribing support program within primary care clinics. The interview guide (see supplemental document) was developed based on previous literature on buprenorphine adoption^9,34,42,44,45^ and research team consensus. We asked participants about their previous experience treating OUD, their willingness to prescribe buprenorphine, and preferences for training on buprenorphine prescribing. The full research team included two social scientists, an infectious disease physician and epidemiologist, an addiction medicine physician, and a biostatistician.

We recruited physicians, PAs, and NPs practicing in rural primary care clinics in Ohio. We also included PCPs who held clinic leadership positions in systems that included rural clinics, some of whom were located in urban counties. Initial participants were identified with the support of the Clinical and Translational Research Unit at Ohio University, and snowball sampling^46^ was used to identify additional interviewees. Specifically, we asked participants for the contact information of additional rural PCPs at the end of the interviews. We also engaged in theoretical sampling, or collecting additional data based on gaps in the developing theoretical framework,^47^ after the first five interviews with the goal of recruiting participants from additional rural regions of Ohio and additional PAs working in primary care. We contacted all participants by email to request participation.

Interviews were conducted concurrently with data analysis,^48^ allowing for refinement of the interview guide, and continued until theoretical saturation was reached. In grounded theory, theoretical saturation refers to the point at which categories are thoroughly described with the data collected and connections between categories are confirmed.^47–49^ The audio-recorded interviews lasted 30 to 60 min, and topics included training background, experience with buprenorphine, and ideas for developing a rural primary care buprenorphine prescribing support program. Participants were compensated with $30 Amazon gift cards. All participants gave their verbal informed consent prior to interviews, and ethics approval was granted by the institutional review board at Ohio University.

### Data Analysis

Interviews were professionally transcribed and uploaded to Dedoose, a cloud-based mixed methods data analysis software,^50^ for analysis. A multi-step process was used to analyze qualitative data. First, an initial codebook was developed using a modified grounded theory, inductive approach.^47^ Four student research assistants (CF, EA, KK, BO) participated in coding after receiving structured training in qualitative data analysis. Each transcript was double coded by two of these research assistants. To ensure internal consistency, all coders coded the first transcript and met to discuss discrepancies. Team members continued to meet biweekly throughout the analysis process to iteratively review the codebook, merge duplicate codes, and discuss emerging themes.

Because several developing themes from the first round of coding were related to inner setting factors that aligned with the implementation climate framework, we undertook a second round of coding using a deductive, framework-based approach.^51^ Starting with the original codebook, we re-analyzed our data and sorted emergent categories from the first round of coding into the six implementation climate subconstructs. We also wrote detailed memos for codes under each subconstruct. Three team members with implementation science expertise (VG, WM, and BF) reviewed the final structure. The goal was to identify which implementation climate subconstructs were perceived to shape buprenorphine prescribing in rural primary care. A Consolidated Criteria for Reporting Qualitative Research (COREQ)^52^ checklist for this study is available (Supplementary documents).

## RESULTS

### Sample Demographics

Twenty-three PCPs participated in the interviews. Of those participants, 5 worked as NPs, 14 worked as physicians, and 4 worked as PAs. Additionally, 14 participants identified as female and 9 male. A total of 13 participants were PCPs working full-time in family medicine, 2 were physicians working full-time in addiction medicine, 1 participant worked full-time in clinical leadership at the state level, 3 participants worked full-time in clinical leadership at the clinic level, 1 participant worked full-time in academic leadership for a PA program, and 2 participants worked in leadership for a family medicine residency program. Twelve participants currently prescribed buprenorphine. Nineteen participants practiced in a rural area, with 2 participants practicing in partially rural and 17 participants practicing in rural counties. Fifteen participants practiced in federally qualified health centers (FQHCs). Participants worked across all five regions of Ohio, with 13 in the southeast region, 5 in the central region, 3 in the northwest region, 1 in the northeast region, and 1 in the southwest region (Table 1).

### Implementation Climate Themes

**Figure 1:**
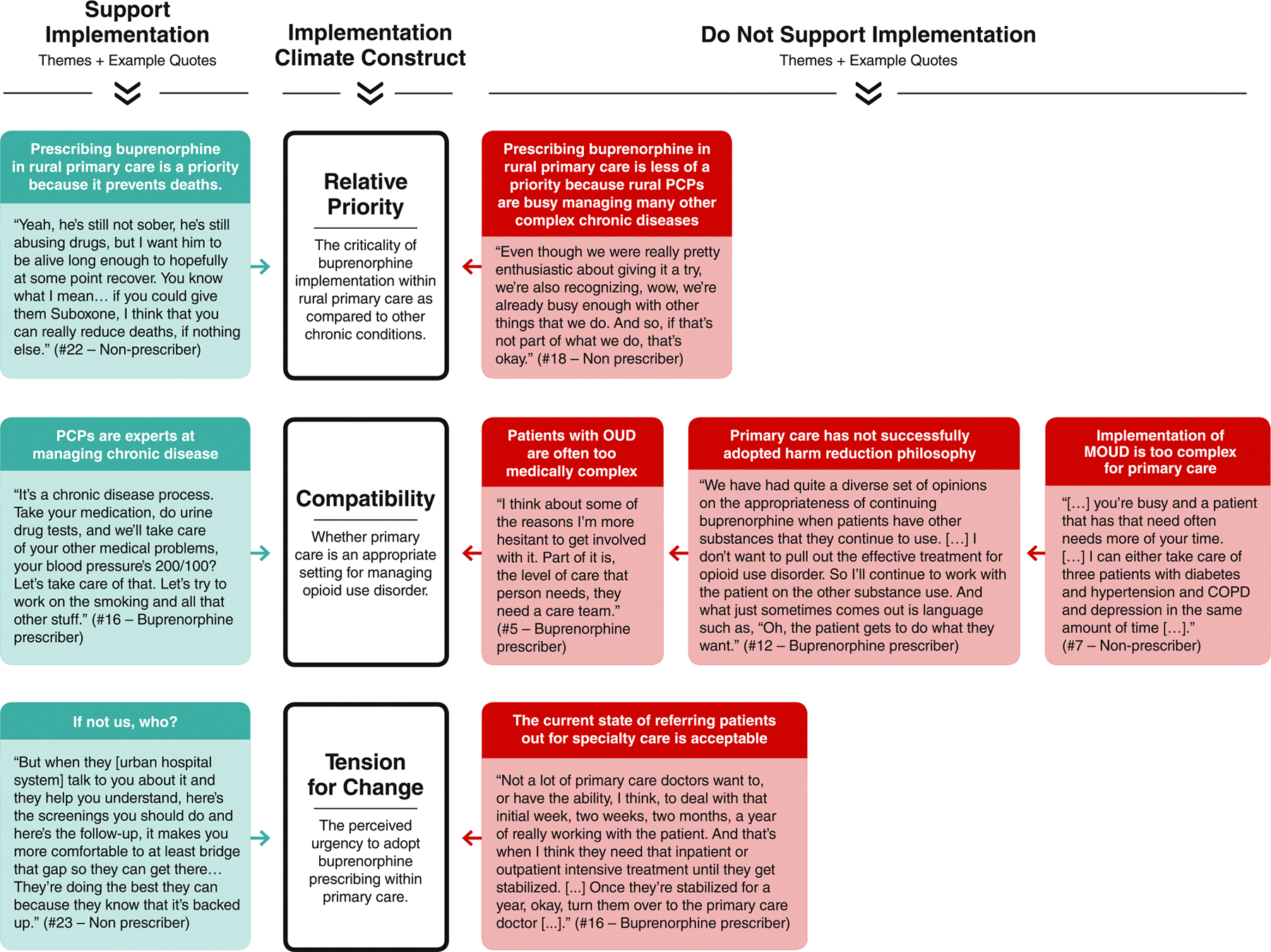
Example quotes by theme and implementation climate construct.

Of the six subconstructs that make up the implementation climate, the data strongly aligned with three (Fig. 1), suggesting that relative priority (perceived importance), compatibility (perceived fit between intervention and existing workflows), and tension for change (perceived urgency for change) are essential factors that shape the implementation climate for buprenorphine prescribing for rural primary care. The remaining three subconstructs, organizational incentives and rewards, goals and feedback, and learning climate, did not align with the categories which emerged from the interviews.

Below we present themes within each of the three aligned subconstructs that both support and do not support implementation of buprenorphine. For each participant, we specified whether they are a current buprenorphine prescriber and listed their training credential.

#### Relative Priority.

The relative priority of prescribing buprenorphine was mixed among participants. The themes were equally split between supporting and not supporting buprenorphine implementation. Some PCP participants believed that buprenorphine could help patients with OUD, but others had never prescribed buprenorphine and felt that other chronic conditions should be prioritized.

##### A. Prescribing Buprenorphine in Rural Primary Care Is a Priority Because It Prevents Deaths

PCP participants frequently described the prevention of overdose deaths as a key reason for implementing buprenorphine. For instance, one participant explained their motivation for prescribing buprenorphine: “There were patients dying left and right, so I did it just out of a sense of responsibility” (#12, physician, prescriber). A PCP, who was not currently prescribing buprenorphine, explained that deaths were a motivator for them to consider prescribing: “The amount of drug abuse that’s going on, somebody’s got to do something” (#11, physician, non-prescriber). Some PCPs noted that regardless of whether patients were engaging in polysubstance use, buprenorphine would still prevent deaths. The opportunity to save lives, even when patients were not in recovery, motivated some PCPs to prioritize OUD treatment within rural primary care.

##### B. Prescribing Buprenorphine in Rural Primary Care Is Less of a Priority Because Rural PCPs Are Busy Managing Many Other Complex Chronic Diseases

Despite the rising number of OUD-related deaths, many PCPs described a sense of conflicting priorities due to the other chronic diseases that they treat in primary care. For instance, one participant stated:

Primary care doctors are busy with diabetes, heart disease, hypertension, getting everybody vaccinated [...] Do you really want to add one more disease process? A guy with COPD, diabetic, [...] you’re going to spend an hour with this person. And that’s not really how it happens in primary care. You end up pushing a lot of that stuff off to somebody else (#16, physician, prescriber).

For some PCPs, competing demands on their time limited their ability to prescribe buprenorphine. Even though many PCPs stated an interest in learning how to prescribe buprenorphine and acknowledged the importance of treating OUD, their existing responsibilities made prescribing buprenorphine difficult to prioritize.

#### Compatibility.

Compared to other subconstructs, the themes under compatibility overwhelmingly did not support implementation. Although many PCP participants felt it was their job to manage chronic disease, they also described barriers, mostly in the inner setting, that made the primary care setting incompatible with treating OUD.

##### A. PCPs Are Experts at Managing Chronic Disease

Responsibility for chronic disease management emerged as a significant theme regarding the mission of rural primary care. Participants frequently noted that PCPs have expertise in managing chronic diseases, such as diabetes, hypertension, and depression, which may support the treatment of OUD in rural primary care as it is a chronic, relapsing condition. As one participant argued, OUD treatment is actually easier than many other chronic diseases:

So I think to the extent that you can emphasize this is manageable and you do have adequate time to do it, it’s no different than a person with diabetes who has very poor blood glucose control and is calling you. Actually, that would probably be more difficult. That would actually be a lot more difficult (#12, physician, prescriber).

Another participant explained that the rural primary care setting is ideal for treating chronic disease, as it supports frequent visits and facilitates longitudinal patient-provider relationships: “Primary care is a great place because you’re having those conversations on a regular basis when it comes to health and well-being and other medical conditions. It comes along with high blood pressure, diabetes…” (#9, physician, prescriber). Another PCP added: “I feel that most primary care providers, after they’re seeing their patients on a frequent basis, they know them better than anybody else” (#15, NP, prescriber).

Another participant argued that while some providers might be new to prescribing buprenorphine, rural PCPs have been among the first to treat other complex chronic diseases, such as depression and chronic kidney disease, with new medications. For example, one PCP described how they have become comfortable providing care that specialists typically provide in an urban setting: “With kidney disease, once you hit a certain stage, you’d be silly not to make sure they had a kidney doctor if they needed dialysis. But I can wait until you’re right up to almost that point before I refer you, versus some people who will refer you immediately when you start to have the smallest bit of decline” (#5, physician, non-prescriber). Finally, participants suggested that buprenorphine is compatible with rural primary care because PCPs are well-equipped to manage co-occurring OUD and mental health conditions, as one participant described: “As we know, SUD often co-occurs with other mental health, and treating that dual diagnosis is super important for recovery. I also treat heroin and alcohol use disorders, as well as methamphetamine disorders, diabetes, COPD, hypertension, wellness exams, immunizations […]” (#3, NP, non-prescriber). PCP’s substantial experience with chronic disease management was seen by some participants as one of their greatest strengths for treating addiction and convinced some PCPs to consider prescribing buprenorphine.

##### B. Patients with OUD Are Often Too Medically Complex

Although rural PCPs were skilled at managing several conditions concurrently, some participants expressed reservations that rural PCPs should manage OUD given that patients often presented with many concurrent conditions. For example, a participant described how she diagnosed a congenital heart defect in one of her patients with OUD that was exacerbated by drug use. They explained the challenges of caring for patients with OUD within a rural residency clinic:

Our population can be a little more... they are a sicker patient in general…I was just charting on a patient earlier this morning, who has a congenital defect in her heart that was never repaired. She has been using drugs and ended up with an infection in one of the valves of her heart, which is complicated by this hole in her heart. She has heart failure. She had to have a kidney removed last year... I’m like, and you want me to treat her substance use disorder? This person needs a village. This is where I’m drawing the line. You need more help than I am able to provide (#5, physician, non-prescriber).

Some rural PCPs felt that managing OUD by themselves would set their practices up for risks if the care provided was not appropriate. They also felt that buprenorphine prescribing in rural primary care was also risky for patients who might not get the level of care they need given the comorbidities that are common in individuals with opioid use disorder. PCPs often expressed discomfort and a lack of confidence treating addiction and instead believed that specialty settings were more appropriate to provide this level of care.

##### C. Primary Care Has Not Successfully Adopted Harm Reduction Philosophy

Some participants also expressed concerns that rural health care organizations still needed to buy into a harm reduction approach to treating addiction for successful implementation of buprenorphine. For instance, a clinic leader described ongoing disagreement about whether a harm reduction, or “low-barrier” approach was appropriate. Acting as an organizational champion, they clashed with their organization’s chief executive officer (CEO), who had an abstinence-aligned approach:

I was really transparent with my CEO. I’m like, ‘This is the direction I need to go with this, and are you going to support me?’ Because otherwise, I’m not going to be able to do this job without his support. He was much more abstinence aligned…there was a lot of conflict happening, and this is because we were so heavy with sanctioning patients (#12, physician, prescriber).

Another PCP, who similarly worked in an organization with many barriers in place to receive buprenorphine described how their colleague became disinterested in prescribing buprenorphine after being exposed to a low-barrier approach during an X-waiver training session. The colleague was in disbelief that treating OUD sometimes requires providers to give patients many opportunities to stop or restart treatment:

I’m kind of conservative, but my nurse practitioner went and did it with me, ultra-conservative. She stayed for the whole thing... She goes, ‘I just don’t think I can do this.’ It was a conflict of interest in her brain. I mean, she just had a hard time with the whole thing (#6, physician, prescriber).

In these organizational climates, some PCPs were unsure if buprenorphine was compatible with the rural primary care setting without an organizational culture that supports harm reduction.

##### D. Implementation of Buprenorphine Is Too Complex for Primary Care

Other participants worried that implementing buprenorphine was too complex for busy primary care clinics, as it would create new demands on staff. One participant shared their experience prescribing buprenorphine in their organization with little support from leadership:

So what I’ve found is that the training’s great. Implementing it is something totally different. What’s your paperwork? What are your consents in your clinic? There is so much you have to develop yourself, that hopefully with any training, they would provide you generic templates that could be made into your own. But I felt like I really had to seek all of that out. We had to develop it all, to implement it (#10, physician, prescriber).

Administrative concerns, such as developing and writing new policies and procedures, navigating legal requirements, new privacy concerns, and training staff, were among the most described. One provider who stated they were very interested in prescribing expressed hesitancy rooted in uncertainty over how they would ever find coverage if they were out of the office. A seemingly basic administrative concern, this matter was not something their rural organization had figured out and was enough to keep them from prescribing:

If I’m capped at how many I can see, I also have to have a partner that can see patients or can cover me when I’m out. And if there’s only two people in town, are they able to pick up that slack also? So there’s all that administrative hoop around it that’s like we’re not doing the best we can with what we have (#7, physician, non-prescriber).

Because so few buprenorphine prescribers currently exist in rural primary care, participants expressed challenges finding a mentor to help them learn this new skill or feared being the only prescriber in their setting with no one else to provide care coverage if they were out of the office.

#### Tension for Change.

The themes under tension for change were equally split between supporting and not supporting implementation of buprenorphine. Many PCP participants felt that the status quo of referring patients out for buprenorphine was not sufficient to meet the needs of their communities. Other PCPs, however, felt that it was acceptable to continue referring patients out to care because specialty addiction treatment settings were more appropriate.

##### A. If Not Us, Who?

Some participants argued that the tension for change in rural primary care is high because there are not enough providers to meet patient needs. Participants described long wait times to see a psychiatrist or addiction specialist in rural areas. One PCP explained their patients are waiting 6 weeks before psychiatry can see them. Another participant, who sees mainly adolescents, said wait times are between 1 and 2 years. After a treatment facility was abruptly closed in 2014, a participant noted the negative impact on health outcomes: “…anytime you close a facility like this, all those people couldn’t get access to medication…we saw a spike in deaths” (#16, physician, prescriber). Given the urgent need for treatment, some participants felt that primary care has a role, at minimum, to serve as a bridge to specialty treatment. Some participants described the tension for change as substantial and described efforts of rural primary care to create policies to extend care quickly in the absence of nearby specialty providers.

##### B. The Current State of Referring Patients Out for Specialty Care Is Acceptable

Some rural PCPs, however, felt uncomfortable starting patients on buprenorphine and felt like it was outside their scope of practice. Instead, these participants suggested that a more appropriate role for primary care would be continuing buprenorphine for patients already stable on the medication, as one participant explained:

I have a patient, he’s been on [MOUD] for two, three years now. He is super stable. He hasn’t had any adjustments. Could he not have to go over there [to a specialist] several times a week? Yeah, probably. So there’s some opening opportunity probably for maintenance people, but not necessarily a lot of tweaking in the future… (#20, PA, non-prescriber).

These participants felt that specialty addiction treatment providers are the most appropriate setting to start buprenorphine and refer patients for care until they are stabilized. From their perspective, expanding buprenorphine access into primary care is not urgent because this setting is not the most appropriate site for acute addiction treatment, or referrals are currently working. As one PCP explained: “if someone’s in a certain situation…I have an immediate lateral referral that I can click a couple of buttons and they can get a quick appointment that we can get them taken care of so that we don’t say, ‘Well, best of luck to you. Here’s some withdrawal medicine. Good luck on this weekend” (#15, NP, prescriber). Although primary care can play a role in buprenorphine access, some participants felt comfortable doing this only once patients were in sustained recovery or felt their referral networks provided sufficient care.

## DISCUSSION

Understanding inner setting factors, such as the implementation climate for adopting buprenorphine, is essential for developing strategies to expand access to this evidence-based medication in rural communities.^34,55,56^ Across the three implementation climate subconstructs, participants described mixed support for adopting buprenorphine in rural primary care, suggesting that multicomponent implementation strategies that target the implementation climate are necessary. PCPs were especially hesitant regarding whether the rural primary care setting was compatible with buprenorphine prescribing, suggesting that strategies are needed to make this setting more conducive to addiction treatment.

Similar to our findings, prior research has found mixed support for expanding buprenorphine in the primary care setting, with PCPs citing concerns such as anticipation of an unmanageable influx of patients, time constraints, limited capacity to absorb new patients, inflexible appointment scheduling processes, and difficulty engaging patients due to community-level stigma.^17,22,55,57–60^ Fewer studies have examined support for expanding buprenorphine in rural primary care, but rural PCPs have cited similar inner setting concerns (e.g., time constraints, a lack of support for complex problems, unsupportive leadership, a lack of space or staff).^34,61–63^

Compared with prior studies, our findings suggest there are additional barriers in rural settings, such as limited organizational support for harm reduction and limited provider time given other chronic disease management. We also identified unique facilitators in rural primary care that may be harnessed through tailored implementation strategies. Emphasizing the high tension for change and the relative priority of buprenorphine, given its potential to save lives in rural primary care, may present a unique opportunity to create buy-in for urgent buprenorphine adoption. Consistent with prior literature, tailored strategies that target the inner setting are also critically needed in rural primary care to help offset persistent prescribing barriers.^64^

It is important to note that three of the subconstructs that make up the implementation climate, organizational incentives and rewards, goals and feedback, and learning climate did not align well with the initial categories that emerged from our data. Although a few participants mentioned financial barriers regarding reimbursement and the time required to provide MOUD, these themes aligned more strongly with compatibility than organizational incentives and rewards. Previous studies, however, have identified organizational incentives and rewards as barriers to prescribing buprenorphine in primary care.^65–67^ Indeed, a recent systematic review found that the institutional environment, including limited staff to support buprenorphine prescribing and limited financial incentives, shaped physician reluctance to provide addiction treatment.^68^ Our results suggest that there might be unique determinants within the rural primary care setting that center on whether the medication is compatible with this practice setting, can be justifiably prioritized among other chronic health conditions, and requires urgent practice change given limited community resources.

### Implementation Climate Strategies to Support Buprenorphine Adoption

Rural PCP participants agreed that implementing buprenorphine is a priority because few actions in primary care could so effectively prevent deaths and infectious diseases from OUD. Nonetheless, some participants struggled with how to effectively prioritize adding buprenorphine to their busy practice. Implementation strategies that harness the strong motivation of individual PCPs to reduce suffering in their communities while establishing organization-level policies, including patient workflows for buprenorphine prescribing, may be critical for increasing buprenorphine adoption within this setting. Prescribing support at the individual level could include brief training and start-up guides for buprenorphine prescribing, and mentorship networks that include other rural PCPs.

Participants also had mixed opinions on whether buprenorphine prescribing was compatible with the rural primary care setting. To support perceived compatibility, implementation strategies could emphasize that addiction treatment follows a chronic disease model^69–71^ and, therefore, is compatible with the primary care setting. In addition, strategies could build efficacy among PCPs and organizational leadership by providing contact with peer PCPs and leaders in rural primary care practice who currently prescribe and support buprenorphine. These efforts could also include information on the effectiveness of harm reduction models,^72–74^ the safety of buprenorphine,^75–77^ management of OUD alongside other chronic conditions, and stigma reduction.^28,78–82^ Finally, implementation strategies that use case managers or social workers^83^ may help support PCPs as they manage patients with OUD and improve care coordination for patients.^84–87^

Because formal treatment settings and specialists were scarce, some PCPs were motivated to prescribe buprenorphine and support their patients who may have trouble gaining access to treatment elsewhere. Still, some PCPs felt hesitant to prescribe altogether or thought that a more acceptable solution would be filling gaps, when necessary, by maintaining buprenorphine prescriptions among patients in stable recovery or by providing bridge prescriptions until patients were able to see a specialist. Implementation strategies that emphasize the critical role that PCPs can fill in preventing opioid overdose^60,88,89^ and infectious disease^90–92^ may be particularly important. Other methods could focus on building confidence that PCPs can not only fill gaps related to buprenorphine access but could actively expand access through screening their existing patient panel and participating in buprenorphine induction.

### Limitations

We collected data from 23 PCPs in Ohio using diverse sampling methods, including convenience sampling (partnering with a statewide health consortium to identify contacts), snowball sampling, and theoretical sampling. Participants came from diverse geographical regions in Ohio, held a variety of credentials, and had different levels of experience prescribing buprenorphine. Still, the findings may not be generalizable to rural communities elsewhere. It is possible that people who agreed to participate were motivated based on strongly held perspectives on buprenorphine. Nonetheless, participants had substantial experience working in rural primary care settings and their perspectives illuminate how rural PCPs weigh expanding access to buprenorphine through primary care. Future studies could use a probability-based sampling method to determine if these findings hold across other rural regions. Our study did not assess the importance of outer setting factors, such as continued regulation of buprenorphine at the state level. Future studies should consider how these factors shape perceptions and willingness to prescribe buprenorphine among rural PCPs.

## CONCLUSION

Rural PCPs have the potential to expand access to buprenorphine, but significant challenges remain. Within rural primary care clinics, the implementation climate is not currently sufficient to support buprenorphine adoption. Specifically, some rural PCPs question whether the primary care setting is compatible with buprenorphine treatment given the significant time spent managing chronic disease and limited organizational support for harm reduction. To address these concerns and build confidence in buprenorphine prescribing, strategies are urgently needed to create a more compatible prescribing environment which aligns buprenorphine treatment with other types of chronic disease management.

## Supplementary Material

COREQ

**Supplementary Information** The online version contains supplementary material available at https://doi.org/10.1007/s11606-024-09260-1.

## Figures and Tables

**Table 1:** Characteristics of Interview Participants

s	Sex	Credential	PCP	Occupation	Prescriber	Region	County classification	FQHC

1	F	NP	Y	Family medicine	N	Southeast	Rural	Y
2	F	MD	Y	Addiction medicine, family medicine, CMO of health system	Y	Central	Urban	Y
3	F	NP	Y	Family medicine	N	Southeast	Rural	Y
4	M	DO	N	Addiction medicine	Y	Southeast	Rural	N
5	F	DO	Y	Family medicine	N	Southeast	Rural	N
6	F	MD	Y	Family medicine	Y	Southeast	Rural	N
7	M	DO	Y	Family medicine residency director	N	Southeast	Rural	N
8	F	MD	Y	Family medicine	N	Northwest	Rural	N
9	F	DO	Y	Family medicine	Y	Southeast	Partially rural	N
10	F	DO	Y	CMO of clinic	Y	Southwest	Rural	Y
11	M	MD	Y	Family medicine	N	Northwest	Rural	Y
12	F	MD	Y	CMO of clinic	Y	Northeast	Urban	Y
13	M	MD	Y	CMO of clinic	Y	Southeast	Rural	Y
14	M	PA	N	Academic leadership	N	Central	Urban	N
15	M	NP	Y	Family medicine	Y	Southeast	Rural	Y
16	M	MD	N	Addiction medicine; public health official	Y	Southeast	Rural	Y
17	F	PA	Y	Family medicine	N	Southeast	Rural	Y
18	M	MD	Y	CMO of clinic	N	Southeast	Partially rural	Y
19	F	MD	Y	Family medicine	Y	Northwest	Rural	Y
20	F	PA	Y	Family medicine	N	Central	Rural	N
21	F	NP	Y	Family medicine	Y	Central	Urban	Y
22	F	NP	Y	Family medicine	N	Southeast	Rural	Y
23	M	PA	Y	Family medicine	Y	Central	Rural	Y

*NP*, nurse practitioner; *PA*, physician assistant; *MD*, doctor of medicine; *DO*, doctor of osteopathic medicine; *CMO*, chief medical officer; Sex: *F*, female; *M*, male; *Y*, yes; *N*, no. County classification is drawn from the Ohio Department of Health, which defines counties as rural, partially rural, or urban.^53^ Regional classification is drawn from *The Five Ohios* by the Ray C. Bliss Institute of Applied Politics at the University of Akron^54^
