# Supplementary material for: The Implementation Climate for Integrating Buprenorphine Prescribing into Rural Primary Care
Source: J Gen Intern Med. 2024 Dec 12;40(12):2879–89. doi: 10.1007/s11606-024-09260-1 (PMC12434468; doi:10.1007/s11606-024-09260-1)
Supplement: Supplementary file 1 — Supplementary file1 (DOCX 26 KB) [file 11606_2024_9260_MOESM1_ESM.docx]

**Consolidated Criteria for Reporting Qualitative Research (COREQ) Checklist**

| **No. Item** | **Item** | **Guide questions/description** | **Comments** | **Page # or Not Applicable (N/A)** |
| --- | --- | --- | --- | --- |
| **Domain 1: Research team and reﬂexivity** |  |  |  |  |
| *Personal Characteristics* |  |  |  |  |
| 1. | Interviewer/facilitator | Which author/s conducted the interview or focus group? | Cheyenne Fenstemaker and Berkeley Franz. | 8 |
| 2. | Credentials | What were the researcher’s credentials? E.g. PhD, MD | Cheyenne Fenstemaker, MA, Elizabeth A. Abrams, MSPH, Katherine King, BA, Benjamin Obringer, BS, Daniel L. Brook, PhD, Vivian Go, PhD, William C. Miller, MD, PhD, Lindsay Y. Dhanani, PhD, and Berkeley Franz, PhD. | 1 |
| 3. | Occupation | What was their occupation at the time of the study? | Cheyenne Fenstemaker, research assistant, Department of Social Medicine and Institute to Advance Health Equity (ADVANCE); graduate student, Elizabeth A. Abrams, medical student, Benjamin Obringer, medical student, Katherine King, children services employee, Daniel L. Brook, medical student, Vivian Go, Professor of Health Behavior, Associate Director of the Center for AIDS Research Social and Behavioral Science Core, and Member of the Institute for Global and Infectious Diseases (IGHID), University of North Carolina at Chapel Hill, William C. Miller, Professor, Department of Epidemiology, University of North Carolina at Chapel Hill, Lindsay Y. Dhanani, Assistant Professor, Human Resource Management, Berkeley Franz, Associate Professor, Community-based Health; Co-director of the Institute to Advance Health Equity (ADVANCE). | 1 |
| 4. | Gender | Was the researcher male or female? | Cheyenne Fenstemaker, Female, Elizabeth A. Abrams, Female, Benjamin Obringer, Male, Katherine King, Female, Lindsay Dhanani, Female, Berkeley Franz, Female. |  |
| 5. | Experience and training | What experience or training did the researcher have? | Cheyenne Fenstemaker, MA in Law, Justice, and Culture; 3 years of experience in qualitative research methods, Elizabeth A. Abrams, MSPH with a focus on social and behavioral interventions and qualitative research theory and methods; several years of experience in qualitative and mixed-methods research,  Katherine King, BA in Sociology; 6 years of experience working in social services with children and families;  Benjamin Obringer, BS in Health Sciences, 1 year of retail pharmacy experience; medical student in second year of training;  Daniel L. Brook, PhD in Epidemiology, MD candidate; Vivian Go, PhD in Health Policy and Management, 20 years of experience in intervention research, implementation science, qualitative methods, and mixed methods; William C. Miller, MD, PhD, 25 years of experience in infectious disease epidemiology with a focus on the intersection of HIV, sexually transmitted infections (STIs), and substance use;  Lindsay Dhanani, PhD in Industrial-Organizational Psychology, extensive training in research methods, 10 years of experience with coding, Berkeley Franz, PhD, medical sociologist with 10 years of research experience in qualitative methods and addiction health services research. She is completing a fellowship in implementation science for addiction research with the Center for Dissemination and Implementation at Stanford University. | 1, 8 |
| *Relationship with participants* |  |  |  |  |
| 6. | Relationship established | Was a relationship established prior to study commencement? | No. | 8-9 |
| 7. | Participant knowledge of the interviewer | What did the participants know about the researcher? e.g. personal goals, reasons for doing the research. | Researcher affiliation, researcher credentials, researcher occupation, and reasons for doing the research. | 8-9 |
| 8. | Interviewer characteristics | What characteristics were reported about the interviewer/facilitator? e.g. Bias, assumptions, reasons and interests in the research topic | Bias, assumptions, reasons and interests in the research topic. | 8-9 |
| **Domain 2: study design** |  |  |  |  |
| *Theoretical framework* |  |  |  |  |
| 9. | Methodological orientation and Theory | What methodological orientation was stated to underpin the study? | Grounded theory; thematic analysis. | 9 |
| *Participant selection* |  |  |  |  |
| 10. | Sampling | How were participants selected? | Convenience, snowball method, and theoretical sampling. | 8 |
| 11. | Method of approach | How were participants approached? | Email. | 8 |
| 12. | Sample size | How many participants were in the study? | 23. | 10 |
| 13. | Non-participation | How many people refused to participate or dropped out? Reasons? | No participants dropped outWe did not collect information on the number of people who refused. | N/A |
| 14. | Setting of data collection | Where was the data collected? | Virtually within homes, and workplaces. | N/A |
| 15. | Presence of non-participants | Was anyone else present besides the participants and researchers? | No. | N/A |
| 16. | Description of sample | What are the important characteristics of the sample? | Participants were from diverse regions of Ohio. Sample also includes different experts on rural primary care. | 8, 10 |
| *Data collection* |  |  |  |  |
| 17. | Interview guide | Were questions, prompts, guides provided by the authors? Was it pilot tested? | Prompts and guides were provided to interviewees upon request. The interview guide was not pilot tested but was refined through the simultaneous data collection and analysis process. | 8, 9 |
| 18. | Repeat interviews | Were repeat interviews carried out? If yes, how many? | No. | N/A |
| 19. | Audio/visual recording | Did the research use audio or visual recording to collect the data? | Yes. Audio and visual recordings were gained through video conferencing technology. |  |
| 20. | Field notes | Were ﬁeld notes made during and/or after the interview or focus group? | No, field research was not conducted. | N/A |
| 21. | Duration | What was the duration of the interviews or focus group? | Interviews were 30 to 60 minutes long. | 9 |
| 22. | Data saturation | Was data saturation discussed? | Yes. | 9 |
| 23. | Transcripts returned | Were transcripts returned to participants for comment and/or correction? | No. | N/A |
| **Domain 3: analysis and ﬁndings** |  |  |  |  |
| *Data analysis* |  |  |  |  |
| 24. | Number of data coders | How many data coders coded the data? | 4 analysts total with 2 analysts coding each transcript. | 9 |
| 25. | Description of the coding tree | Did authors provide a description of the coding tree? | The authors lay out a thematic structure related to the Implementation Climate for buprenorphine prescribing in rural primary care. The full code book is available upon reasonable request. | 9-10 |
| 26. | Derivation of themes | Were themes identiﬁed in advance or derived from the data? | Derived from data. | 9-18 |
| 27. | Software | What software, if applicable, was used to manage the data? | Dedoose version 9.0.46. | 9 |
| 28. | Participant checking | Did participants provide feedback on the ﬁndings? | Yes. In interviews we provided participants with preliminary findings for feedback. | 9 |
| 29. | Quotations presented | Were participant quotations presented to illustrate the themes/ﬁndings? Was each quotation identiﬁed? e.g. participant number | Yes. | 11-18 |
| 30. | Data and ﬁndings consistent | Was there consistency between the data presented and the ﬁndings? | Yes. | 11-18 |
| 31. | Clarity of major themes | Were major themes clearly presented in the ﬁndings? | Yes. | 11-18 |
| 32. | Clarity of minor themes | Is there a description of diverse cases or discussion of minor themes? | Yes. | 11-18 |

Developed from: Tong A, Sainsbury P, Craig J. Consolidated criteria for reporting qualitative research (COREQ): a 32-item checklist for interviews and focus groups. International Journal for Quality in Health Care. 2007. Volume 19, Number 6: pp. 349 – 357
